# Megalocytiviruses in ornamental fish: A review

**DOI:** 10.14202/vetworld.2020.2565-2577

**Published:** 2020-11-30

**Authors:** Che Azarulzaman Che Johan, Sandra Catherine Zainathan

**Affiliations:** Department of Fisheries and Aquaculture, Faculty of Fisheries and Food Science, University Malaysia Terengganu, Terengganu, Malaysia

**Keywords:** clinical signs, detection, *Megalocytivirus*, ornamental fish, risk factors

## Abstract

Iridoviruses, especially megalocytiviruses, are related to severe disease resulting in high economic losses in the aquaculture industry worldwide. The ornamental fish industry has been affected severely due to *Megalocytivirus* infections. *Megalocytivirus* is a DNA virus that has three genera; including red sea bream iridovirus, infectious spleen and kidney necrosis virus, and turbot reddish body iridovirus. *Megalocytivirus* causes non-specific clinical signs in ornamental fish. Cell culture, histology, immunofluorescence test, polymerase chain reaction (PCR) assay, and loop-mediated isothermal amplification assay have been used to diagnose megalocytiviruses. Risk factors such as temperature, transportation (export and import), and life stages of ornamental fish have been reported for the previous cases due to *Megalocytivirus* infections. In addition, other prevention and control methods also have been practiced in farms to prevent *Megalocytivirus* outbreaks. This is the first review of megalocytiviruses in ornamental fish since its first detection in 1989. This review discusses the occurrences of *Megalocytivirus* in ornamental fish, including the history, clinical signs, detection method, risk factors, and prevention measures.

## Introduction

Ornamental fish are known to be affected by *Megalocytivirus* [1-5], which can cause systemic infections in a wide variety of freshwater and marine fish [6], including amphibians [7]. *Megalocytivirus* is an important genus of fish viruses in the Iridoviridae (the Iridoviruses) family [8]. The Iridoviridae family is divided into five genera, which include *Chloriridovirus*, *Iridovirus*, *Lymphocystivirus*, *Megalocytivirus*, and *Ranavirus*. *Megalocytivirus* is the most recently added genus [9,10]. Megalocytiviruses are large icosahedral DNA viruses measuring 120-200 nm in diameter [11] and have a large single linear dsDNA genome [12]. *Megalocytivirus* isolates exhibit relatively few genetic differences and have been divided into three major groups based on genetic sequencing data. These groups are represented by infectious spleen and kidney necrosis virus (ISKNV), red sea bream iridovirus (RSIV), and turbot reddish body iridovirus (TRBIV) [13]. The most frequently identified megalocytiviruses are RSIV and ISKNV [14]. Song *et al*. [15] evaluated 48 Asian and Australian *Megalocytivirus* isolates with regard to their geographic locations and genetic variations in the major capsid protein (MCP) gene. Based on the results, they developed a phylogenetic tree that divided the 48 isolates into three distinct clusters based on genotype. One of these clusters (genotype I) is widely distributed among several Asian countries, including 13 isolates from Korea, nine isolates from Japan, one isolate from Thailand, one isolate from China, and one isolate from the South China Sea [15]. In contrast, the other two genotypes have smaller host ranges and are locally distributed [15]. Genotype II megalocytiviruses infect freshwater fishes from Southeast Asia and Australia, whereas genotype III megalocytiviruses primarily infect flatfish in China and Korea [15].

The first outbreak of *Megalocytivirus*-induced disease was recorded in the Japanese cultured red sea bream (*Pagrus major*) in 1990 and was designated as RSIV disease [8]. In 2001, the complete genome sequence of ISKNV was determined using the next-generation sequencing technique VIDISCA-454, where sequences of an unknown virus were detected in the serum of diseased fish. The near-complete genome sequence of the virus was determined, which showed a unique genome organization and low levels of identity to known members of the Iridoviridae. Based on the homology of a series of putatively encoded proteins, the virus was identified as a novel member of the *Megalocytivirus* genus in 2001 [8], and in the following year in Japan, *Megalocytivirus* was identified in ornamental fish, African lampeye *Aplocheilichthys normani*, and dwarf gourami *Colisa lalia* imported from Singapore [16]. Next, in 2005, the identification of genotypes within the species of *Megalocytivirus* we made [17], and in 2006, genetic vaccines for RSIV were generated from DNA vaccines against RSIV infection in fish using the MCP gene and an open reading frame (ORF) containing a transmembrane domain [18].

In 2006, it was understood that global trade of ornamental fish may facilitate the spread of *Megalocytivirus* and enable the emergence of disease in new host species in distant biogeographic regions [19]. In the same year, an outbreak of iridovirus was reported among various fish species in Taiwan. These megalocytiviruses showed high homology (97% identity) to RSIV with the presence of unidentical nucleotide sequences [20]. In 2008, a phylogenetic tree revealed three clusters: Genotype I, including nine Japanese isolates, 13 Korean isolates, one Chinese isolates, one Thai isolate, and one South China Sea isolate; genotype II, including five freshwater fish isolates in Southeast Asian countries and Australia; and genotype III, consisting mainly of flatfish isolates in Korea and China. This suggests that viruses belonging to genotype I were widely distributed among various fish species in many Asian countries [15]. Furthermore, in 2009, a detailed study of ISKNV infection in an imported ornamental fish was conducted. Phylogenetic analyses using sequences from a portion of the DNA polymerase, MCP, and ATPase genes of *Banggai cardinalfish iridovirus* in *Pterapogon kauderni* with the presence and systemic distribution of enlarged virus-infected cells, demonstrated a close relationship with ISKNV [21].

This review is intended to provide an update on the prevalence of megalocytiviruses, including the history, clinical signs, detection method, risk factors, and prevention measures, which will of interest to aquaculturists that are involved in ornamental fish farming and trading around the world.

## History of Diagnosis and Cases of *Megalocytivirus* Infection in Ornamental Fish

Many cases have reported on the infection of *Megalocytivirus* in ornamental fish. In 1989, the presence of numerous virions as large aggregates within many cells in chromide cichlid *Etroplus maculatus* was revealed. The polyhedral particles measured approximately 180-200 nm and had a round central core [22]. The structure, size, and cytoplasmic assembly of the virion classified it within the Iridoviridae family [22]. In 1990, a juvenile angelfish (*Pterophyllum scalare*) was bought from a local pet shop in Canada and introduced into a tank that contained matured angelfish from the same shop. The juvenile angelfish was found dead 60 h after it was transferred into the tank. A histological test found massive necrosis and hemorrhage of the hematopoietic tissue in the kidney and spleen and necrosis of the pancreas [23]. Anderson *et a*l. [24] reported that dwarf gourami, *C. lalia* imported from Singapore, displayed systemic amoebiasis in intestinal and peritoneal lesions. Electron microscopy observation demonstrated the presence of iridovirus-like virions. A batch of juvenile angelfish (*P. scalare*) bred in the United Kingdom died within 2 days of purchase [25]. The spleen of the two fish was sampled, and the histopathology result showed the presence of numerous virions in large groups in the cell cytoplasm. The structure and size of the virions suggested that they were Iridoviruses, yet that was not confirmed until much later. In 2001, there was an outbreak caused by Iridoviruses in tropical ornamental fish farms in Israel. A systemic viral infection in *Trichopodus trichopteru*s*, Trichopodus leerii*, and swordtail *Xiphophorus hellerii* has occurred in endothelial cells that become hypertrophic, and an outbreak of lymphocystis in freshwater angelfish *P. scalare* and gourami was also reported [26].

ISKNV-like viruses, African lampeye iridovirus, and dwarf gourami iridovirus (DGIV) were detected in freshwater African lampeye (*A. normani*) and dwarf gourami (*C. lalia*), which were imported into Japan in 2002 [16]. *A. normani* was cultured in freshwater ponds in Indonesia Island of Sumatra and *C. lalia* was cultured in freshwater ponds in Malaysia before tropical fish wholesalers from Singapore exported it to Japan. The histology result for *A. normani* showed many inclusion body-bearing cells (IBCs) and necrotic cells in the pulp from spleen. Meanwhile, the spleen of *C. lalia* displayed many early-stage IBCs, matured IBCs, and necrotic cells in the pulps, and the kidney showed many matured IBCs in the hematopoietic tissue. Gibson-Kueh *et al*. [27] examined four species of tropical freshwater ornamental fish, including dwarf gourami (*C. lalia*), pearl gourami (*T. leeri*), Siamese fighting fish (*Betta splendens*), and angelfish (*P. scalare*). The samples were derived from newly dead fish. The histological results showed that all of the affected fish demonstrated basophilic and hypertrophied cells in various organs. Petty and Fraser [28] found iridovirus in freshwater angelfish that showed an enlarged abdomen and abnormal body in a high mortality occurrence at the farm.

In 2005, the International Committee on Taxonomy of Viruses accepted a new genus known as *Megalocytivirus* under the Iridoviridae family based on the appearance of enlarged cells that were observed on histological examination of infected fish [29]. Many cases of outbreak caused by megalocytiviruses in marine and freshwater ornamental fish were reported after 2005. Jeong *et al*. [30] revealed outbreaks of ISKNV in ten freshwater ornamental fish species (*T. leeri, T. microlepis, C. lalia, Xiphophorus maculatus, X. helleri, Poecilia sphenops, Lebistes reticulatus, Astronotus ocellatus, Hyphessobrycon innesi*, and *Pterophyllum eimekei*). The fish were sampled from wholesalers in Korea and importers from China and Singapore. All of the ornamental fish species tested were positive for iridovirus through two-step polymerase chain reaction (PCR). Paradise fish (*Macropodus opercularis*) imported from Indonesia [31], common platy, pearl gourami, zebrafish, swordtail, ram cichlid from major ornamental fish breeding states in peninsular Malaysia [32], platys *X. maculatus* from Australia [33], angelfish *Pterophyllum altum* imported from Colombia, platys *X. maculatus* of unknown origin [5], *X. hellerii*, *X. maculatus*, *P. sphenops*, *T. trichopterus* [4], *Poecilia reticulata, T. leeri, Apistogramma ramirezi* [3] from Southern Malaysia, zebrafish *Danio rerio* from a research facility in Spain [34], and three marine ornamental fish, Banggai cardinalfish *P. kauderni* [21], orbiculate batfish *Platax orbicularis* [35], and majestic angelfish *Pomacanthus navarchus* [2] have reported positive for megalocytiviruses. Other ornamental fish species from around the world that have been detected for megalocytiviruses infection since 1989 until 2019 are listed in Table-1 [1-5,15,16,20-22,25-27,30-32,34,36-41].

**Table-1:** Ornamental fish with known susceptibility to Megalocytiviruses around the world from year 1989 to 2019.

Family	Species	Common name	Region of origin/country	Year	References
Apogonidae	*Pterapogon kauderni*	Banggai cardinalfish (Marine)	Banggai archipelago through Bali or Singapore	2003-2005	[21]
Arapaimidae	*Arapaima gigas*	Arapaima	Brazil	2015	[2]
Cichlidae	*Astronotus ocellatus*	Oscar	USA Possibly South east Asia Malaysia/Sri Lanka/Singapore Thailand	Early 1990s 2004-2006 2013 2016-2018	[28] [36] [37] [1]
	*Etroplus maculatus* *Mikrogeophagus* *ramirezi/Apistogramma ramirezi* *Pterophyllum scalare*	Orange chromide Ram cichlid (blue ram) Freshwater angelfish	Singapore Malaysia/ Sri Lanka/ Singapore Malaysia Malaysia Not specific	1989 2010-2012 Not specific 2017 1990	[22] [37] [32] [3] [25,28]
Cichlidae	*Pterophyllum* *scalare*	Freshwater angelfish	United Kingdom Singapore Malaysia/Sri Lanka/ Singapore Brazil Thailand	1997 1992-2000 2009 2015 2016-2018	[25] [27] [37] [2] [1]
	*Pterophyllum altum*	Altum angelfish, deep angelfish, or Orinoco angelfish	Columbia	2014	[5]
	*Apistogramma cacatuoides* *Laetacara curviceps* *Pelvicachromis kribensis*	Cockatoo dwarf cichlid Flag acara Kribensis	Malaysia/Sri Lanka/Singapore Malaysia/Sri Lanka/Singapore Malaysia/Sri Lanka/Singapore	2010-2012 2010 2012	[37] [37] [37]
	*Tropheus duboisi*	White spotted cichlid	Malaysia/Sri Lanka/Singapore	2012	[37]
	*Cichlasoma* sp. *Symphysodon* sp.	- Discus	Thailand Thailand	2016-2018 2016-2018	[1] [1]
Characidae	*Paracheirodon innesi*	Neon tetra	Korea/Singapore/China	2014	[30]
Characidae	*Metynnis argenteus* *Moenkhausia costae*	Silver dollar Tetra fortune Brazil	Malaysia/Sri Lanka/ Singapore	2010 2015	[37] [2]
Cyprinidae	*Carassius auratus* *Brachydanio* *albolineatus* *Danio rerio*	Gold fish Pearl danio Zebrafish	Brazil Brazil Malaysia Spain	2015 2015 Not specific 2015	[2] [2] [32] [34]
Cobitidae	*Misgurnus anguillicaudatus*	Pond loach	Brazil	2015	[2]
Ephippidae	*Platax orbicularis*	Orbiculate batfish (Marine)	Indonesia	2010	[38]
Helostomatidae	*Helostoma temminckii*	Kissing gourami	USA Singapore, Sri Lanka, and Thailand	Early 1990s 2011	[28] [35]
Hemiodontidae Loricariidae	*Hemiodus gracilis* *Hypostomus plecostomus*	Slender Hemiodus Suckermouth catfish	Brazil Brazil	2015 2015	[2] [2]
Labridae Nothobranchiidae	*Labroides dimidiatus* *Fundulopanchax gardneri*	Doctor fish Blue lyretail	South east Asia Malaysia/Sri Lanka/ Singapore	1995 2012	[39] [37]
Osphronemidae	*Betta splendens* *Colisa lalia/Trichogaster lalius*	Siamese fighting fish Dwarf gourami	Singapore Thailand Singapore	1992-2000 2016-2018 1988	[27] [1] [35]
			USA Singapore Malaysia via Singapore South east Asia Singapore Indonesia and Thailand	Early 1990s 1992-2000 2000 2004 2004 2011	[28] [27] [16] [20] [30] [35]
	*Colisa labiosa/* *Trichogaster labiosa*	Thick lipped gourami	Asia Thailand	2006 2011	[19,20] [35]
	*Trichogaster trichopterus/* *Trichopodus trichopterus*	Three spot gourami/ Blue or Gold gourami	USA	1992	[28,40]
Osphronemidae	*Trichogaster trichopterus/Trichopodus trichopterus*	Three spot gourami/blue or gold gourami	Indonesia, Singapore and Thailand Brazil Malaysia	2011 2015 2016	[35] [2] [4]
	*Trichogaster leerii*	Pearl gourami	USA Singapore South east Asia Singapore Indonesia Brazil Malaysia Malaysia Israel	Early 1990s 1992-2000 2003 2004 2011 2015 2017 Not specific Not specific	[28] [27] [20] [30] [35] [2] [3] [32] [26]
	*Trichogaster microlepis*	Moonlight/silver gourami	Singapore Brazil Malaysia Malaysia Israel	2004 2015 2017 Not specific Not specific	[30] [2] [3] [32] [26]
	*Trichogaster microlepis*	Moonlight/silver gourami	Singapore	2004	[30]
Osphronemidae	*Macropodus opercularis*	Round tail paradise fish	Indonesia Brazil	2006 and 2008 2015	[31] [2]
Poeciliidae	*Aplocheilichthys normani* *Poecilia latipinna*	Norman’s (African) lampeye Sailfin molly	Indonesia via Singapore Malaysia/Sri Lanka/ Singapore Thailand Israel	1998 2012 2016-2018 Not specific	[16] [37] [1] [26]
	*Xiphophorus hellerii*	Swordtail	Malaysia/Sri Lanka/ Singapore Malaysia Thailand Israel Malaysia	2012 2016 2016-2018 Not specific Not specific	[37] [4] [1] [26] [32]
	*Xiphophorus maculatus*	Southern platy fish	Malaysia/Sri Lanka/Singapore Not specific Brazil	2012 2014 2015	[37] [5] [2]
Poeciliidae	*Xiphophorus maculatus*	Southern platy fish	Malaysia	2016 and 2017	[3,15]
			Thailand Israel Malaysia	2016-2018 Not specific Not specific	[1] [26] [32]
	*Poecilia reticulata*	Guppy	South east Asia Korea/Singapore/China Brazil Thailand Malaysia	1995 2014 2015 2016-2018 2017	[41] [30] [2] [1] [3]
	*Poecilia sphenops*	Molly (or sphenops molly)	Korea/Singapore/China Malaysia	2014 2016	[30] [4]
	*Xiphophorus variatus*	Variable platy	Thailand	2016-2018	[1]
*Pomacanthidae*	*Pomacanthus navarchus*	Blue girdled angelfish/Majestic angelfish (Marine)	Brazil	2015	[2]

## Common Clinical Signs of *Megalocytivirus* in Ornamental Fish

*Megalocytivirus* infection is known to cause non-specific clinical signs in infected ornamental fish, similar to clinical signs that are common for other diseases [13,42]. The most common external clinical signs observed due to *Megalocytivirus* infection (Table-2) [1-5,13,15,16,21-23,25,28,31,38,42] and mortality [5,13,35,42] have been reported in many studies. An unusual white fecal was produced by *P. kauderni* from California before the fish died [21]. *Megalocytivirus*-infected fish have been reported to be asymptomatic or apparently healthy [1,3,4,30,34,35]. The absence of specific clinical signs in ornamental fish has made it difficult for farmers to detect *Megalocytivirus*; therefore, *Megalocytivirus*-positive fish often exported overseas during the asymptomatic carrier stage [3,4]. Histological analysis is needed to observe the pathological signs caused by *Megalocytivirus* (Table-3) [1,4,16,21,26-28,31,38,43-47]. *Megalocytivirus*-infected fish are known to develop IBCs, which are mostly found in spleen, kidney, and liver [16,22,23,29,37], considering the pathognomonics of infection with megalocytiviruses [8,48]. In addition, the histological results showed that the *Megalocytivirus*-like infection in juvenile angelfish *P. scalare* demonstrated massive necrosis and hemorrhage in the kidney and spleen and necrosis in the pancreas [26] and intestine of dwarf gourami *C. lalia* [24]. The production of basophilic hypertrophied cells [13,21,27,29,38] is the most common symptom observed in *Megalocytivirus*-infected fish. Commonly affected sites include the submucosa and tunica propria of the intestine and stomach [16] instead of the spleen, liver, and kidney [38]. An experimental challenge of ISKNV in zebra fish (*Danio rerio)* showed erratic swimming patterns, lingering near the surface of the water, hemorrhages, and scale protrusion. Histological results found necrosis of tissue and enlarged cells in the kidney and spleen [49].

**Table-2:** The common external clinical signs of *Megalocytivirus* infection observed in ornamental fish.

External clinical signs	References
Lethargy	[2,13,21,31,38]
Skin lesion	[38]
Abnormal swimming pattern in the water	[1,2,15,25,42]
Loss of appetite	[2,5,15,21]
Pale or darkening body coloration	[1,3,15,16,21,22,42]
Distended body	[4,13,25,28]
Ulceration	[2,42]
Pale gills anemia	[4,16,22,25]
Hemorrhages	[1,15,21,23,28]
Abnormal body posture	[28]

**Table-3:** The pathological findings of *Megalocytivirus* in affected fish.

Pathological signs	References
Hypertrophic cells	[26,27]
Splenomegaly (enlargement of the spleen)	[1,16,31,38]
Hepatomegaly (enlargement of liver)	[1,4,38]
Necrosis	[16,21,28]
Congestion	[43-46]
Heterochromatin of infected cell	[47]

## Laboratory Diagnosis of *Megalocytivirus* Infection in Ornamental Fish

Many studies have diagnosed *Megalocytivirus* infection using histopathology, cell culture, immunofluorescent antibody techniques, PCR, and more recently, loop-mediated isothermal amplification (LAMP) [50]. In ornamental fish, cell culture was initiated using imported freshwater dwarf gourami [24] and marine Banggai cardinalfish (*P. kauderni*) [21], but these efforts were unsuccessful due to the difficulty of isolation and propagation in cell cultures [51], lack of validated cell culture techniques [52], and the requirement for a longer duration [53]. The viral titrate and infectivity decreased on serial passages in many cell lines, although many common fish cell lines supported their growth [54,55]. Only few strains of megalocytiviruses could be diagnosed by cell culture, including RSIV in tilapia heart cell culture. RSIV can be cultured in tilapia heart cell culture using minimum essential medium, 10% fetal bovine serum, and 50 μg gentamicin/ml, and incubating the cultures at 28°C [40]. Fortunately, in 2003, the *Megalocytivirus*-like viral particles isolated from dwarf gourami originated from Singapore and were found to be able to grow in BF-2 cells at 20°C in Scotland [27]. The cell culture lines that were used to detect *Megalocytivirus* are listed in Table-4 [16,46,54,56-67].

**Table-4:** The cell culture of *Megalocytivirus* strain in fish.

Cell lines	Strain of *Megalocytivirus*	References
Grunt fin cells (GF cells)	RSIV and ISKNV	[58]
	*Megalocytivirus*	[16,45]
	Rock bream iridovirus	[56]
Mandarin fish fry (MFF-1 and MFF-8-C1 cells)	ISKNV and RSIV-like	[54,57]
Caudal fin of red sea bream (CRF-1 cells)	RSIV	[58]
Dorsal fin tissue from red sea bream (PI-RSBF-2 cell line)	RSIV	[59]
Brain tissue of orange spotted grouper (GBC-4 cells)	Giant seaperch iridovirus (GSIV-K1)	[60]
Embryonic bastard halibut tissue (FEC)	Turbot reddish body iridovirus	[61]
Brown marbled grouper fin and heart tissue (bmGF-1 and bmGH cells)		[62,63]
Turbot fin tissue (TF cell line)		[64]
Stone flounder liver (SFL cells)		[65]
BF-2 or GF cells	Grouper spawner iridovirus	[66]
KRE cells	Taiwan grouper iridovirus	[67]

The first immunofluorescence test was used against RSIV using monoclonal antibodies in 1995 [68]; however, early or latent infection cannot be detected using this technique [53]. Among the advantages of this technique include the fact that it does not cross-react within megalocytiviruses (RSIV or SBIV) or ranaviruses (epizootic hematopoietic necrosis virus or GIV) [69]; it requires a short time (within 2 h); and it provides precise results [50]. This assay is also suitable for the diagnosis of ISKNV [8]. Immunofluorescence has been used to detect ISKNV in experimentally infected zebrafish, *D. rerio*, in 2008. The ISKNV-infected hypertrophic cells were present in different organs of moribund zebrafish, including the spleen, kidney, liver, gill, esophagus, gut, and muscle, as detected using the viral protein VP23R, which is encoded by the ORF23R of ISKNV and specifically localized on the plasma membrane of the ISKNV-infected cells [49].

PCR is one of the most commonly used methods for detecting the viral DNA and MCP gene of *Megalocytivirus* [37]. There are different types of PCRs, but the most common method used to detect *Megalocytivirus* in ornamental fish are conventional and real-time PCRs. The conventional PCR detects the presence or absence of the DNA/RNA virus in infected fish. The most preferred method is real-time PCR, because it is more sensitive compared to conventional PCR [12], and specific DNA regions of species can be determined using specifically designed probes [70,71]. In contrast, conventional PCR is widely used in studies (Table-5) [1-5,16,30-35,37,72].

**Table-5:** Polymerase chain reaction assays used to detect *Megalocytivirus* in ornamental fish.

Species	Common name	Assay type	References
*Aplocheilichthys normani*	African lampeye	Single step	[16]
*Lebistes reticulatus*	Guppies	Nested	[3,30]
*Poecilia sphenops*	Molly	Nested	[4,30]
*Xiphophorus hellerii*	Swordtail	Nested	[4,30]
*Trichogaster leeri*	Pearl gourami	Nested	[3,30]
*Macropodus opercularis*	Paradise fish	Nested	[2,31]
*Danio rerio*	Zebra fish	Single step	[32,34]
*Pterophyllum altum*	Angelfish	Single step	[5]
*Colisa lalia*	Dwarf gourami	Single step	[16]
		Nested	[30]
*Hyphessobrycon innesi*	Neon tetras	Nested	[30]
*Pterophyllum eimekei*	Angelfish	Nested	
*Trichogaster microlepis*	Silver gourami	Nested	
*Xiphophorus maculatus*	Platy	Real-time PCR and nested	[1,33]
		qPCR and nested	[35]
		Nested	[2,4,30,37]
		Single step	[5]
		qPCR	[72]
*Astronotus ocellatus*	Oscars	Real-time PCR and nested	[1]
		Nested	[30,37]
		qPCR	[72]
*Mikrogeophagus ramirezi*	Ram cichlid	Nested	[3]
		Single step	[32]
		qPCR	[72]
*Poecilia latipinna*	Sailfin molly	Real-time PCR and nested	[1]
		qPCR and nested	[35]
		Nested	[37]
		qPCR	[72]
*Trichopodus trichopterus*	Gold gourami	Nested	[2,4]
		Real-time PCR and nested	[1]
*Helostoma temminckii*	kissing gourami	qPCR	[72]
*Mikrogeophagus altispinosus*	Bolivian ram	qPCR	
*Sphaerichthys sp*	Chocolate gourami	qPCR	
Species undefine	Discus	qPCR	
Species undefine	Archerfish	qPCR	
*Carassius auratus*	Goldfish	Nested	[2]
*Moenkhausia costae*	Tetra fortune	Nested	
*Brachydanio albolineatus*	Pearl danio	Nested	
*Misgurnus anguillicaudatus*	Pond loach	Nested	
*Hypostomus plecostomus*	Suckermouth catfish	Nested	
*Hemiodopsis gracilis*	Slender hemiodus	Nested	
*Pomacanthus narvachus*	Blue girdled angelfish	Nested	

**Figure-1:**
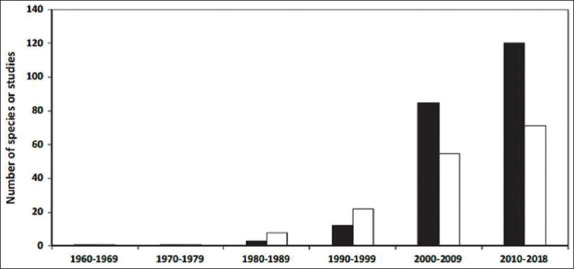
The results of genotype and quantitative using peptide nucleic acid probe in different diseased fish between 2012 and 2018 in Korea [73].

Real-time PCR is more sensitive than conventional PCR for the detection of fish viruses. Rimmer *et al*. [52] demonstrated the sensitivity of real-time PCR for the detection of DGIV. The sensitivity was approximately 3-4 logs greater compared to the Office International des Epizooties reference PCR protocol. Real-time PCR was used to analyze the mRNA level of ISKNV replicated in zebrafish [49]. In 2020, a new assay was discovered for the detection and genotyping of *Megalocytivirus* instead of DNA hybridization, which is known as peptide nucleic acid (PNA)-based real-time PCR assay in Korea [73]. It has more suitable hybridization traits that allow for a difference in the melting temperature, even by a single nucleotide mismatch, and discrimination of the genotypes of *Megalocytivirus* in a single simple assay [73]. Four PNA probes labeled with the respective fluorescence at their 3′ ends were designed as reporter molecules. The study was tested in rock bream (*Oplegnathus fasciatu*s). The result for this new method was similar to the sequencing analysis of *Megalocytivirus-*infected fish and artificial samples (Figure-1).

LAMP assay is another method used to detect megalocytiviruses [74]. Basically, the LAMP assay uses four primers to identify six different sequences of the target DNA [69,75]. Compared to PCR [76], LAMP is a simple, rapid (within an hour) [50,77,78], specific, and cost-effective [50] nucleic acid amplification method that can be performed at temperatures between 60°C and 65°C [79,80]. LAMP utilizes self-sustained sequence replication and can synthesize a large amount of DNA [76]. LAMP assay has been tested to detect megalocytiviruses in infected fish, including guppies, gourami, angelfish, swordtail fish, and platy [81]. This assay has been developed for TRBIV [78], RSIV [82], and ISKNV [83]. LAMP assay is very sensitive, as it can detect as little as ten copies of the megalocytiviral DNA target [78,82,83]. Subramaniam *et al*. [32] used acridine orange to improve the visual quality of the LAMP assay for ISKNV.

## Risk factors

### Temperature

High water temperatures had been shown to enhance the multiplication of *Megalocytivirus* [12,13,60,84]. Yanong and Waltzek [13] reported that *Megalocytivirus* could survive at temperatures ranging from 20°C to 32°C. A review report from the Australian Department of Agriculture [6] stated that the relationship of infectivity of *Megalocytivirus* is inversely proportional to the temperature and time [6]. ISKNV can remain infective even when isolated and kept under −70°C for >8 months and 40°C for 30 min, but the virus was inactivated when it was isolated and kept under 50°C for 30 min [6]. This will give the optimum temperature for the virus to remain active throughout the year and could be one of the factors that lead to disease outbreak [13,85]. Limited studies have been conducted to evaluate the effect of temperature on *Megalocytivirus* in ornamental fish specifically, but some studies have been conducted on ISKNV in food fish; generally. ISKNV is one of the most isolated *Megalocytivirus* strains in ornamental fish [6,13].

For example, in 2002, in China, the effect of temperature on the pathogenicity of ISKNV infection was conducted [86] in mandarin fish (*Siniperca chuatsi*). The fish was injected intraperitoneally with isolated ISKNV. The result showed that total mortality occurred at 25°C and 34°C, and the natural outbreaks of ISKNV in mandarin fish occurred at 20-32°C. In another study in the same region, two-step PCR showed the percentage of ISKNV-like viruses detected in different seasons (22.1% winter, 27.8% summer, 34.0% autumn, and 17.0% spring) and proved that water temperature is a key condition for ISKNV outbreaks [16]. In other regions, such as Malaysia and Indonesia, *Megalocytivirus* have been observed in water temperatures ranging from 28°C to 32°C annually [85], with the highest mortality occurring at 27°C [87].

#### Transportation

Transportation and translocation of ornamental fish could be the cause of the spread of *Megalocytivirus* [1,31]. Importation is one of the potential vectors of infection [41,86]. In South Korea, paradise fish *M. opercularis* imported from Indonesia was detected with *Megalocytivirus* [31]. In Thailand, a study was conducted on the translocation of *Megalocytivirus* contributing to its spread, and the result showed that the virus could spread by movement of the fries [1]. The transmission of *Megalocytivirus* in ornamental fish based on findings of the previous studies include horizontal transmission through contaminated water, cohabitation with infected fish, ingestion of infected excreta, and cannibalism [6,20]. So, far, no vertical transmission of *Megalocytivirus* has been reported until now.

#### Life stages

The life stages of fish have been reported as one of the risk factors for *Megalocytivirus* infection. Early detection of *Megalocytivirus* in fish has been detected at the juvenile stage. Anderson *et al*. [24] reported that juvenile to young adult dwarf gouramis were infected with *Megalocytivirus*. Juvenile cichlids (freshwater angelfish in the United Kingdom and orange chromide cichlids from Canada) also have been infected by *Megalocytivirus* [22,25]. These results indicate that the juvenile stage is more susceptible to infection of this virus in ornamental fish.

#### Translocation

Translocation of megalocytiviruses through transmission from infected ornamental fish to food fish also has been studied. Experimental transmission trials were undertaken to test the hypothesis that the outbreak of Murray cod (*Maccullochella peelii peelii*) fingerlings in Australis could have arisen through the introduction of a virus with ornamental gouramis imported from the Southeast Asia. The study showed that 90% mortality was induced in Murray cod fingerlings by cohabitation with dwarf gouramis, *C. lalia* [20]. Approximately 125-130 nm icosahedral virions were observed in lesions by histopathological analysis, and the DNA sequencing confirmed 99.9-100% homology between the MCP and ATPase nucleotide sequences of DGIV and ISKNV, as revealed by PCR [20]. These findings confirm that Murray cod is highly susceptible to a *Megalocytivirus* present in ornamental fish imported from Southeast Asia.

Moreover, a study conducted in South Korea on translocation of genus *Megalocytivirus* (pearl gourami iridovirus [PGIV]-SP) from freshwater ornamental fish, pearl gourami *T. leeri*, has shown that it can infect marine fish rock bream, *O. fasciatus*. The virus was isolated from infected pearl gourami and was injected intraperitoneally to the rock bream. The result showed 100% mortality of rock bream after 2 weeks of IP injection. The infection of PGIV-SP isolated from pearl gourami was confirmed by PCR assay [88]. This result proves that megalocytiviruses can be translocated from freshwater into marine habitats, where it can establish a new infection pathway if the virus is released into the natural environment.

In 2019, sixty ornamental fishes were collected from different areas of northern, central, and western Thailand. All were tested by PCR analysis. The result showed that the ornamental fishes were infected by *Megalocytivirus*. The translocation of the virus was analyzed using Chi-square test statistics, which proved that the translocation of *Megalocytivirus* from an unknown or *Megalocytivirus*-positive origin was related to disease occurrence [1].

## Prevention and Control Measures

Vaccine development is important to minimize the infection of megalocytiviruses [89] and to increase the survival rate of fish. To date, vaccine development in aquaculture has focused on food fish only. Moreover, no vaccines have been developed for megalocytiviruses infection in ornamental fish. Due to the small size of the ornamental fish species, it is difficult to produce and administer vaccines to them without causing stress or side effects. There are various methods of fish vaccination, such as trough injection (intramuscular and intraperitoneal) [90], immersion, and oral [90,91]. Ornamental fish are mostly small in size compared to food fish, so the best vaccination for ornamental fish would be through immersion or the oral method.

Oral vaccination can be applied by mixing the vaccine with the feed [89,92]. Two techniques of oral vaccination that has been used for fish include bio-encapsulation and micro-encapsulation [89], but the most suitable and easiest method to use for ornamental fish is bio-encapsulation. Micro-encapsulation is used for sensitive antigens. The bio-encapsulation technique uses life feed, such as *Artemia nauplii*, rotifers, and copepods incubated in a vaccine suspension, which was then fed to the fish [92]. This method can be applied at the early stage of the fish life cycle (the juvenile stage) [93], as it can prevent early infection of megalocytiviruses in ornamental fish. The advantage of the oral vaccination is that it can be used easily in a large scale of small fish without stressing ornamental fish, and it can also lower the labor cost of vaccination development [93].

In addition, immersion vaccinations also can be used for ornamental fish. The immersion method includes bath and dip techniques [92]. As for the immersion method, the fish is normally submerged in water containing the diluted vaccine. The skin and gill absorb the suspended antigens from the vaccine. At that point, the fish would be protected from the live pathogens due to the activation of specialized cells that are present in the skin and gill epithelium, such as antibody-secreting cells [93]. The dip technique is more suitable for ornamental fish compared to the bath technique due to the fact that the technique uses a high concentration of vaccine solution and the fish are immersed in a very short time, approximately 30 s. However, for the bath technique, the fish are immersed approximately 1 to several hours in a low concentration of vaccine solution [92]. Ornamental fish farmers are able to use the immersion technique, as this method can be used for a large-scale vaccination of a high number of ornamental fish, and it is suitable for all sizes of ornamental fish, reduces the labor cost for vaccination implementation, and finally, it can reduce stress [93] on the ornamental fish.

In ornamental fish in China, zebrafish were used to study the infection of ISKNV. The vaccine was administered by IP injection to the zebrafish. The results showed that recombinant zebrafish interferon 1 was used to protect zebrafish from infection by ISKNV after 6 h of injection; however, after 24 h of injection, the result was not significant enough to provide protection for zebrafish against ISKNV [94]. Studies on the pathogenic mechanisms of megalocytiviruses in ornamental fish must be done in the future to increase scientific knowledge of the ornamental fish immune system and the pathogenic and virulence mechanisms so that vaccines can be developed for ornamental fish infected megalocytiviruses.

In contrast, for aquacultured fish, the earliest vaccine was developed to prevent RSIV infection (another strain of *Megalocytivirus*) in Japan [95]. The commercialized injectable vaccine using formalin-inactivated RSIV showed that the survival of red seabream increased 19.2% in a field test [95]. This vaccine has been tested *in vitro* in other fish species, including yellow tail (*Seriola quinqueradiata*; 7% mortality), amberjack (*Seriola dumerili*; 5% mortality), kelp grouper (*Epinephelus moara*; 0% mortality), and striped jack (*Pseudocaranx dentex*; 42% mortality) in Japan, and glycogen storage disease type IV in humpback grouper *Cromileptes altivelis* from Bali Island, Indonesia, showed 0% mortality [96,97]. Zhang *et al*. [98] constructed a vaccine against *Megalocytivirus* using the genes of rock bream iridovirus isolate 1 from China. The result showed that pCN86, a plasmid that expresses an 86-residue viral protein, is an effective DNA vaccine that may be used in aquaculture diseases. Furthermore, P247 and P523, the *Megalocytivirus* immunogens, were selected for use in the DNA vaccine (pCN247 and pCN523). An immune analysis showed that the vaccines induced the production of specific serum antibodies, causing the generation of cytotoxic immune cells and specific memory immune cells that responded to secondary antigen stimulation and upregulated the expression of genes involved in innate and adaptive immunity [99]. In Korea, Oh *et al*. [100] tested the RSIV vaccine in rock bream *O. fasciatus* at a low rearing temperature (≤18°C) and showed that the infection was not expressed as a disease.

Good husbandry and biosecurity measures [11,89] could decrease the chances of *Megalocytivirus* infection as follows. Producers should quarantine any new fish in a separate building or area before the introduction of new fish in the pond, follow appropriate biosecurity protocols, separate incoming fish groups based on origin, use separate equipment, separate unhealthy fish from healthy fish, and disinfect water sources prior use [15]. In addition, farmers must improve their disease monitoring effort, and greater action can be taken by authorities to prevent the disease from spreading [11].

Viral diseases can be controlled by eradication [101] using chemical substances. Essentially, eradication is used to eliminate the disease from a defined geographical area [102]. The principal of eradication is to ensure that the prevalence of a disease is reduced to zero, with or without the use of intervention measures, and that there is total cessation of disease transmission in the defined geographical area [101]. As recent as 2019, in Australia, a study was conducted to evaluate the effect of disinfectants on inactivating ISKNV [101] in ornamental fish. The result showed that the protocols to disinfect ISKNV included using soiling conditions by heating at 65°C for 20 min; immersed in 1000 ppm sodium hypochlorite solution at a pH 3 or pH 11 for 30 min for pH treatment (0.1 M sodium hydroxide (NaOH) or 11.65 M hydrochloric acid (HCl) was added to ISKNV to adjust the pH level using digital pH meter and after 30 min at the desired pH, NaOH or HCl was added to adjust the pH to 7.3); and soaking in 1% Virkon™ or 650 ppm benzalkonium chloride for 10 min for chemical treatments by buffer exchange (a regenerated cellulose 30,000 molecular weight cut-off membrane centrifuge device was prepared by pre-rinsing the membrane with ultrapure water (Amicon Ultra-15 Ultracel, Merck Millipore). Then, the buffer exchange device was loaded with the treated challenge inoculum and centrifuged at 4000 *g* for 5 min at 4 °C) [101]. The quantitative PCR (qPCR) was assessed for the quantity of ISKNV DNA before and after the disinfection method [101]. The disinfection methods and practical application must be explored in future studies, because different regions around the world have different geographical characteristics; thus, different approaches have to be implemented according to regional factors. A cost-effective measure must be developed to lower the cost of farm maintenance so that early outbreaks of megalocytiviruses in the farm can be prevented.

## Conclusion

This is the first review of occurrence of *Megalocytivirus* in ornamental fish since its first detection in 1989. *Megalocytivirus* causes non-specific clinical signs, persists as a carrier in some species, and causes mortalities in other species. The disease is of great concern, as it can cause high mortalities in ornamental fish farming, thus leading to severe economic losses in the aquaculture industry. The disease could be caused by many factors, such as temperature, transportation, and life stages. The future of this industry should lead toward the development of vaccines and other treatment methods for *Megalocytivirus* as an approach to decrease infection cases around the world.

## Authors’ Contributions

CACJ contributed to the original draft and conception of the specific review. SCZ contributed to the review, editing, and supported in supervision. CACJ and SCZ worked on the final approval of the version to be published. All authors read and approved the final manuscript.
